# Quantitative proteomic analysis of plasma membranes from the fish pathogen *Saprolegnia parasitica* reveals promising targets for disease control

**DOI:** 10.1128/spectrum.00348-24

**Published:** 2024-06-18

**Authors:** Hugo Mélida, Lisa Kappel, Sadia Fida Ullah, Vincent Bulone, Vaibhav Srivastava

**Affiliations:** 1 Division of Glycoscience, Department of Chemistry, CBH School, KTH Royal Institute of Technology, AlbaNova University Centre, Stockholm, Sweden; 2 College of Medicine and Public Health, Flinders University, Bedford Park, South Australia, Australia; Universidade de Sao Paulo, Sao Paulo, Brazil

**Keywords:** *Saprolegnia*, disease control, proteomics, microdomains, plasma membrane

## Abstract

The phylum Oomycota contains economically important pathogens of animals and plants, including *Saprolegnia parasitica*, the causal agent of the fish disease saprolegniasis. Due to intense fish farming and banning of the most effective control measures, saprolegniasis has re-emerged as a major challenge for the aquaculture industry. Oomycete cells are surrounded by a polysaccharide-rich cell wall matrix that, in addition to being essential for cell growth, also functions as a protective “armor.” Consequently, the enzymes responsible for cell wall synthesis provide potential targets for disease control. Oomycete cell wall biosynthetic enzymes are predicted to be plasma membrane proteins. To identify these proteins, we applied a quantitative (iTRAQ) mass spectrometry-based proteomics approach to the plasma membrane of the hyphal cells of *S. parasitica*, providing the first complete plasma membrane proteome of an oomycete species. Of significance is the identification of 65 proteins enriched in detergent-resistant microdomains (DRMs). *In silico* analysis showed that DRM-enriched proteins are mainly involved in molecular transport and β-1,3-glucan synthesis, potentially contributing to pathogenesis. Moreover, biochemical characterization of the glycosyltransferase activity in these microdomains further supported their role in β-1,3-glucan synthesis. Altogether, the knowledge gained in this study provides a basis for developing disease control measures targeting specific plasma membrane proteins in *S. parasitica*.

**IMPORTANCE:**

The significance of this research lies in its potential to combat saprolegniasis, a detrimental fish disease, which has resurged due to intensive fish farming and regulatory restrictions. By targeting enzymes responsible for cell wall synthesis in *Saprolegnia parasitica*, this study uncovers potential avenues for disease control. Particularly noteworthy is the identification of several proteins enriched in membrane microdomains, offering insights into molecular mechanisms potentially involved in pathogenesis. Understanding the role of these proteins provides a foundation for developing targeted disease control measures. Overall, this research holds promise for safeguarding the aquaculture industry against the challenges posed by saprolegniasis.

## INTRODUCTION

Oomycetes form a large class of widely distributed eukaryotic microbial organisms. Many oomycete species are pathogens of animals or plants and have serious environmental and economic impact (1). Species from the genus *Saprolegnia* are likely present in all freshwater ecosystems, both natural and aquaculture environments, and include pathogens of amphibians, crustaceans, fish, and insects (2, 3). As a result of intensive aquatic farming and the banning of malachite green, one of the most effective anti-oomycete chemicals, *Saprolegnia* populations have increased dramatically. More specifically, populations of *Saprolegnia parasitica*, the causal agent of the fish disease saprolegniasis, have resulted in losses in the aquaculture industry estimated at tens of millions of dollars annually (2). Consequently, there is an urgent need to identify new, efficient management methods for this devastating disease.

Oomycete cells are surrounded by a polysaccharide-rich cell wall matrix (4). This semi-rigid and highly dynamic structure gives cells the mechanical strength to withstand changes associated with environmental processes, including alterations in osmotic pressure. Although the cell wall serves as a protective “armor” in oomycetes, it has been largely overlooked as a target for disease control in this class of organisms. Interestingly, many enzymes that play important roles in cell wall biosynthesis are associated with the plasma membrane (PM). Recently, differential expression of genes (DEG) encoding for membrane-bound proteins was reported after treatment of *S. parasitica* with the fungicides metalaxyl, bronopol, or copper sulphate (5, 6). For example, four genes encoding β-1,3-glucan synthases were among the identified DEGs in response to copper sulphate (6). It follows that proteomic studies characterizing PM enzymes across different oomycete species may assist the development of species-specific disease control measures. The PM forms a physical interface between the cytoplasm and the extracellular environment and is considered one of the cell’s most interactive and dynamic supramolecular structures (7). Its strategic position allows for involvement in many biological processes, such as metabolite and ion transport, gaseous exchanges, endocytosis, defense against pathogens, cell differentiation, and proliferation (7). Various biochemical and analytical approaches have characterized the PM proteome in different types of organisms, including yeast, plants, and animals (8–10). In the case of oomycetes, the few proteomic approaches available have essentially focused on the genus *Phytophthora*, which includes some of the most infamous plant pathogenic species (11–17). To date, only two proteomic studies investigating oomycete PM have been completed. The first focused exclusively on the plant pathogen *Phytophthora infestans*. Although the authors reported 27 membrane-associated proteins, very few contained transmembrane domains or other motifs that would facilitate binding to the plasma membrane (18). The second study examined the oomycete *Saprolegnia monoica* and included a biochemical analysis of functional PM microdomains (19). In eukaryotic cells, some PM proteins are enriched in lateral lipid patches frequently referred to as “membrane (lipid) rafts,” which form microdomains within the membrane (20). These microdomains are involved in regulating specific PM-associated biological processes and are typically enriched in sphingolipids, sterols, and phospholipids composed of saturated fatty acids (20, 21). The lipid composition of these rafts provides limited resistance to non-ionic detergents (e.g., Triton X-100), a feature which has been experimentally exploited to isolate detergent-resistant microdomains (DRMs) (21, 22). It follows that DRMs provide an excellent system to isolate, identify, and study groups of proteins and lipids that naturally interact and form specialized functional units within the PM. Furthermore, biochemical characterization of DRMs is useful to better understand the protein composition and biological activity of subdomains of the PM. PM microdomains have been characterized in yeast, plants, and animals (10, 21, 23–25). It has been shown that DRM proteins are primarily associated with processes including transport, signal transduction, and response to stress (24, 26–28). In poplar DRMs, cell wall polysaccharide synthases were first observed in a biochemical activity assay (29) followed by the identification of some of these enzymes by mass spectrometry (24). In addition, similar observations were made from biochemical assays examining DRMs isolated from the oomycete *Saprolegnia monoica* (19). Together, these data suggest that DRMs are associated with cell wall biosynthesis.

Despite *Saprolegnia parasitica* being recognised as a major threat to the aquaculture industry, the protein composition of its PM and associated microdomains remains uncharacterized. To address this knowledge gap, we have used a quantitative proteomics strategy to analyze the PM proteome of *S. parasitica*, with a particular focus on proteins enriched in DRMs. *In silico* and biochemical analyses of the DRM-enriched proteins support their involvement in molecular transport and β-1,3-glucan synthesis. Disruption of these processes would have important implications for the infection stages that result to saprolegniasis, highlighting the potential for exploitation in disease management strategies. To our knowledge, this is the first quantitative proteomics analysis characterizing a *Saprolegnia* PM proteome.

## MATERIALS AND METHODS

### Strain and culture methods

All analyses were performed on the mycelial cells of *S. parasitica* Coker 1923 (CBS 223.65). *S. parasitica* was maintained on potato dextrose agar (Sigma-Aldrich), and mycelia used in all the experiments were grown for 4 days at 25°C in liquid Machlis medium (30).

### Isolation of PM and DRM fractions

**Fig 1:**
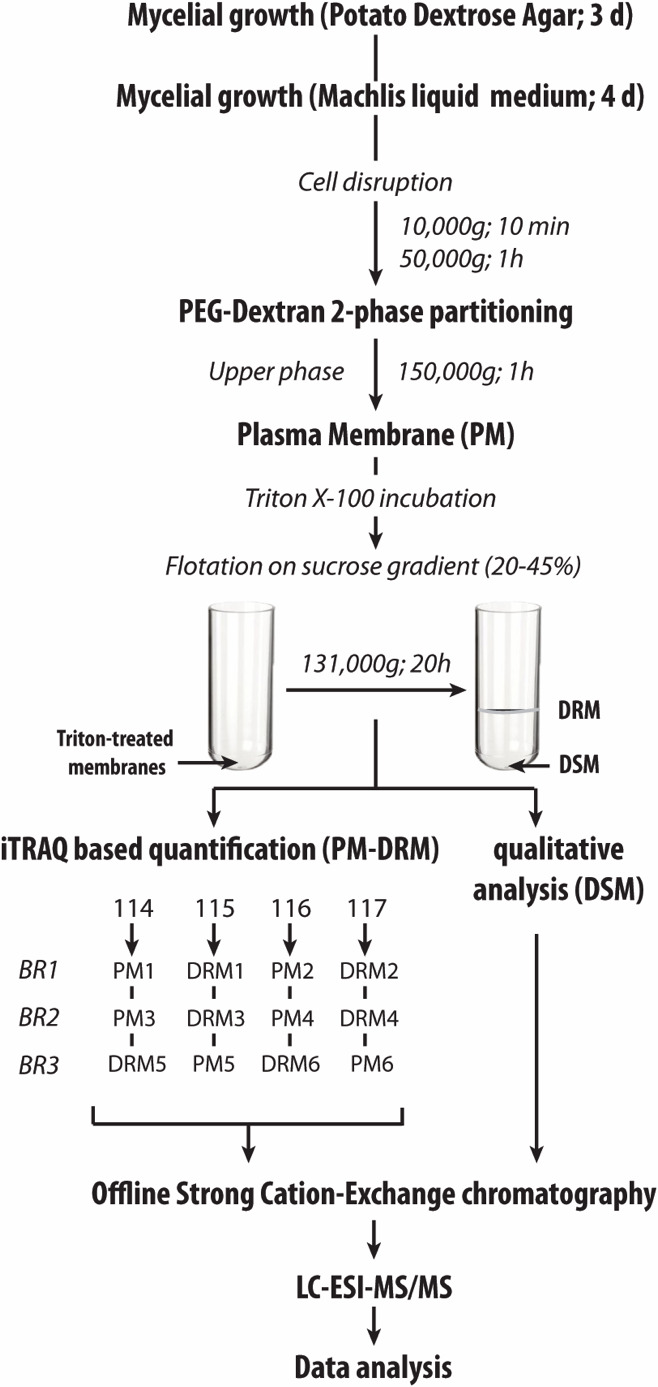
Experimental setup. PM, plasma membrane; DRM, detergent-resistant microdomains; DSM, detergent-soluble membranes; iTRAQ, isobaric tags for relative and absolute quantitation.

The PM and DRMs were isolated as previously described (19) and summarized in Fig. 1. All the steps were performed at 4°C. First, the cells were harvested, washed with water, and dried under vacuum. The PM was purified from the microsomal fraction by two-phase partitioning based on poly(ethylene glycol) 3350 (Sigma-Aldrich) and dextran T-500 (Pharmacosmos). The isolated PM was then resuspended in MOPS buffer (0.05 M, pH 7.0), and the protein concentration was measured using the Bradford dye-binding assay (Bio-Rad).

For isolation of the DRMs, the PM sample was mixed with Triton X-100 (final concentration 1%) [detergent-to-protein ratio = 15:1 (wt/wt)] and incubated for 30 min at 4°C. A sucrose solution was then added to a final concentration of 46%. The preparation was overlaid with a continuous sucrose gradient (45%–15%) and centrifuged at 131,000 × *g* for 20 h at 4°C using a swinging-type rotor (SW27; Beckman). The DRM fraction, which was visible as a white floating band, was collected. The Triton-solubilized proteins sedimented at the bottom of the tube (DSM). Both DRM and DSM fractions were resuspended in MOPS buffer (0.05 M, pH 7.0), and protein concentrations were determined as described above. A total of three biological replicates were prepared. The PM and DRM samples were used for quantitative iTRAQ experiments, whereas the DSM samples were used directly for in-solution digestion, as shown in Fig. 1.

### In-solution digestion, iTRAQ labeling, and strong cation exchange fractionation

Protein extraction and partial hydrolysis were completed as previously described (20). Briefly, protein fractions (PM, DRM, and DSM; 100 µg each) were solubilized in 0.05 M triethylammonium bicarbonate (TEAB) containing 1% sodium deoxycholate (SDC; Sigma). Reduction and alkylation of cysteine residues were completed using tris(2-carboxyethyl)phosphine and methyl methanethiosulfonate, respectively. The proteins were then partially hydrolyzed for 16 h at 37°C in the presence of 5 µg of trypsin in 50 mM TEAB. Tryptic digests were acidified by adding trifluoroacetic acid (TFA) to a final concentration of 0.5%, mixed, and centrifuged to remove SDC. The supernatant was transferred to a new tube and dried under vacuum. Peptides originating from the PM and DRM samples were labeled with different iTRAQ reagents according to the manufacturer’s instructions (114, 115, 116, and 117; AB SCIEX). The iTRAQ-labeled peptides of PM and DRM samples were pooled and dried under vacuum. For the remaining two biological replicates, the iTRAQ labels of the peptides were inverted, and the samples were treated as described above (see Fig. 1).

Both the dried peptides from the DSM and the mixed iTRAQ-labeled PM and DRM samples were loaded on a Nuvia S cartridge (Bio-Rad; prepared according to the manufacturer’s instructions) followed by elution at pH 3.0 with consecutive salt plugs of ammonium formate (30, 50, 80, 100, 125, 150, 175, 200, 225, 250, 275, 300, 325, 350, and 400 mM, in 20% acetonitrile). The eluent from each salt plug was dried, and the peptides were purified on a PepClean C-18 column (Thermo Scientific).

### Nano-LC-ESI MS/MS analysis

Peptide analysis was performed by liquid chromatography-electrospray ionization-tandem mass spectrometry (LC-ESI-MS/MS) using a nanoACQUITY Ultra performance liquid chromatography system coupled to a Q-TOF mass spectrometer (Xevo QTof, Waters Corporation) as described by Srivastava et al. (24). The dried peptides were resuspended in 0.1% TFA, loaded onto a C18 trap column (2G nanoACQUITY UPLC, Symmetry 180 µm × 20 mm, 5 µm; Waters), and washed with 1% (vol/vol) acetonitrile and 0.1% (vol/vol) formic acid at 15 µL/min for 10 min. The samples were eluted from the trap column and separated on a C18 analytical column (75 µm × 150 mm, 1.7 µm; Waters) at 300 nL/min, using 0.1% formic acid as solvent A and 0.1% formic acid in acetonitrile as solvent B, in a gradient as follows: 0.1%%–10% B (0–5 min), 10%%–25% B (5–185 min), 25%%–45% B (185–203 min), 45%%–85% B (203–205 min), 85% B (205–213 min), and 85%%–0.1% B (213–215 min). The eluted peptides were sprayed into the mass spectrometer using a 2.3-kV capillary and 40-V cone voltage. The acquisition of MS/MS spectra was performed with automated data-directed switching between the MS and MS/MS modes using the instrument software (MassLynx V4.1 SP4). The five most abundant signals of a survey scan (400–1,600 *m/z* range, 0.9-s scan time) were selected by charge state, and the collision energy was applied accordingly for sequential MS/MS fragmentation scanning (50–1,800 *m/z* range, 0.9 s scan time).

### Data processing, protein identification, and quantification

An Automated Proteomics Pipeline (APP) (31) was used to analyze the mass spectrometry data. The raw data files were first processed by Mascot Distiller (version 2.4.3.2, MatrixScience). Three search engines, an in-house Mascot server (version 2.4.3.2, MatrixScience), Myrimatch [(32); version 2.1.120], and X!Tandem [(33); version 2011.12.01.1, LabKey, Insilicos, ISB, Seattle, WA], were used to search the *Saprolegnia* protein database [(27); *S. parasitica* CBS 223.65; http://www.broadinstitute.org/] with the following settings: trypsin-specific digestion with two missed cleavages allowed, peptide tolerance of 100 ppm, fragment tolerance of 0.2 Da, methylthio on Cys, iTRAQ 4-plex for peptide N-term, and K as fixed modifications, and, in the variable mode, iTRAQ 4-plex on Y and oxidized methionine was used. For DSM samples, all search parameters were identical but without the iTRAQ-related steps. Results from all search engines were validated by PeptideProphet (34) and ultimately combined using iProphet (35) and ProteinProphet (36). For quantitative analysis, iTRAQ reporter ion intensities were extracted using the TPP tool Libra (http://tools.proteomecenter.org/wiki/index.php?title=Software:Libra#In_a_nutshell) with isotopic correction factors provided by the iTRAQ reagent manufacturer. Normalization of iTRAQ channels and calculation of final protein ratios were performed as previously described (24). A concatenated target-decoy database-search strategy was also used to check the false-positive rate (<1%).

Peptide sequences were exported for each protein, with a protein and peptide probability cutoff of 0.95. Peptides matching two or more proteins (shared peptides) were excluded from the analysis. Proteins with no unique peptides (i.e., only identified by shared peptides) were also excluded. A protein was considered as identified if it contained at least one unique peptide. Only proteins identified by two or more unique peptides were used for quantification.

The statistical analysis of the quantitative data were performed as described by Ross et al. (37). Briefly, each protein’s DRM/PM ratio was calculated for each of the three biological replicates (Tables S2 through S4) and log2 transformed to obtain a normal distribution. The values were then normalized to the median log values, and global means and standard deviations were calculated for each biological replicate. Proteins whose average ratios fell outside a standard deviation of ±1 from the global mean in at least two of three biological replicates were significantly enriched.

### Gene Ontology, prediction of topology, and post-translational modifications

Gene Ontology analysis of identified proteins was performed using Blast2GO Version 2.6.4 (38). Gene Ontology codes were obtained by performing a BLASTp search against the non-redundant database with an expectation value maximum of 1*E*^−3^ and a high scoring segment pair length cutoff of 33. Protein sequences were then mapped and annotated according to the following parameters: pre-eValue-Hit-Filter of 1*E*^−6^, pro-Similarity-Hit-Filter of 15, Annotation CutOff of 55, and GO Weight of 5. Transmembrane domains (TMDs) were predicted using the HMMTOP program (39). The glycosylphosphatidylinositol (GPI) modification and palmitoylation sites were analyzed using the PredGPI predictor and CSS-Palm 4.0 (using the highest thresholds) algorithms, respectively. SignalP 4.1 (40) was used to predict signal peptide sites. Protein Molecular Weight and Isoelectric Point Calculators were used to determine the protein theoretical molecular masses and isoelectric points (pI).

### Assay of carbohydrate synthase activities

Glycosyltransferase activities were assayed as previously described (41). Aliquots containing 15 µg of protein from the PM, DSM, and DRM fractions were used directly in the assays. In some experiments, Triton X-100 was added to the reaction mixtures to final concentrations ranging between 0.2 and 2% (wt/vol). Detergent-solubilized PM samples were obtained after 30 min of continuous stirring in the presence of 1% (wt/vol) Triton X-100 or 0.5% (wt/vol) Chaps and centrifugation (150,000 × *g*, 1 h, 4°C) of the mixtures. 1,3-Glucan synthase activity was measured by mixing the enzyme samples with adequate volumes of the reaction mixture to obtain final concentrations of 7.5 mM PIPES-Tris (pH 6.0), 1 mM UDP-glucose, and 0.16 µM UDP-D-[U-^14^C]glucose (PerkinElmer; 250 mCi mmol^−1^). For chitin synthase activity assays, the final concentrations in the reaction mixture were 10 mM Tris-HCl (pH 7.4), 10 mM MgCl_2_, 20 mM *N*-acetylglucosamine, 1.25 µg mL^−1^ trypsin, 0.475 mM UDP-*N*-acetylglucosamine, and 434 nM UDP-*N*-acetyl-D-[U-^14^C]glucosamine (American Radiolabeled Chemicals; 300 mCi mmol^−1^). The samples were incubated at 25°C for 1 h, reactions were terminated by the addition of ethanol, and following an overnight incubation at −20°C, the precipitated radioactive polysaccharides were quantified by liquid scintillation counting (41).

### Protein domain and phylogenetic trees

Conserved protein domains were identified using the Conserved Domain Database (42) and PROSITE (43) tools and represented using DomainDraw (44). Protein sequences for phylogenetic analysis of *S. parasitica* glucan synthases were retrieved from NCBI (https://www.ncbi.nlm.nih.gov/) and compared with sequences from *Saccharomyces cerevisiae* retrieved from SGD [(45), https://www.yeastgenome.org], from *Arabidopsis thaliana* retrieved from TAIR [(46), https://www.arabidopsis.org], and from the Joint Genome Institute for all other sequences. The phylogenetic tree was constructed with Mega11 [(47), version 11.0.13] using the Neighbor Joining method and 1,000 bootstrap rearrangements to stabilize clades.

## RESULTS

**Fig 2:**
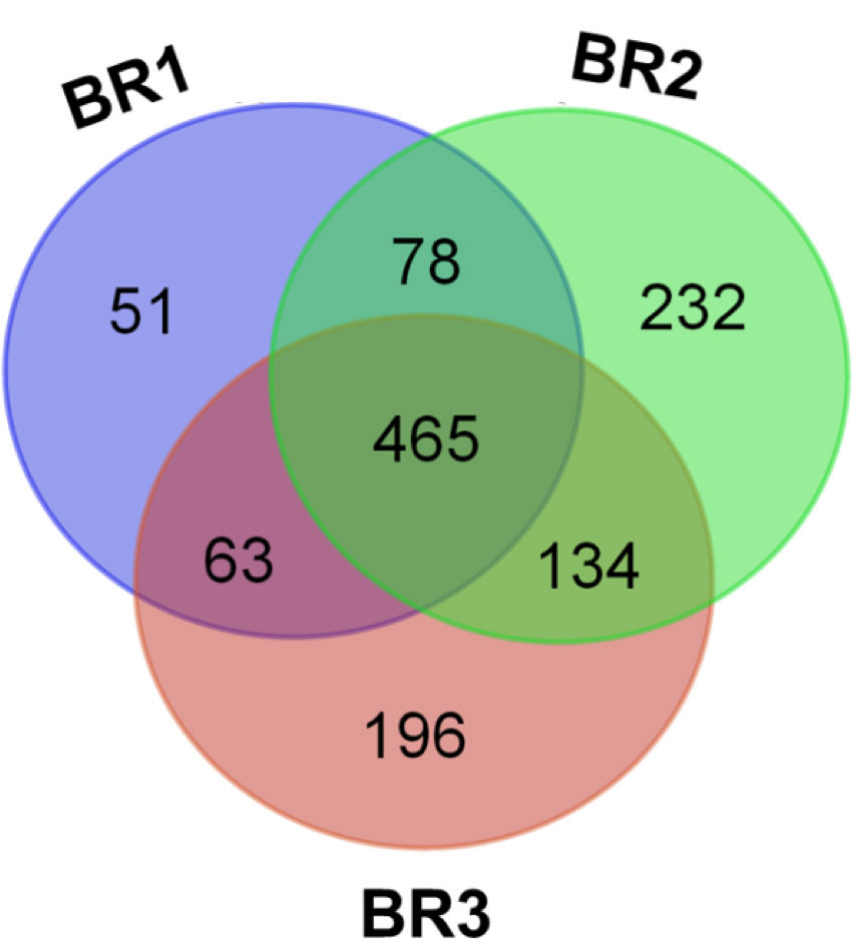
Qualitative proteomic analysis of PM (plasma membrane) and DRM (detergent-resistant microdomains). The Venn diagram represents the number of proteins identified from in-solution digestion and MS/MS analysis of PM and DRM fractions for each of the three biological replicates (BR). In total, 657, 909, and 858 proteins were identified by MS/MS analysis of BR1, BR2, and BR3, respectively.

In this work, we profiled proteins from the PM of the pathogenic oomycete *S. parasitica* with particular focus on DRMs. We used quantitative analysis to examine the type of proteins enriched in these regions to improve our understanding of the functions associated with these specialized microdomains. A total of 1,219 unique proteins were identified from three biological replicates of PM and DRM samples (Fig. 2). Of those identified, 465 were present in all three replicates (Fig. 2). Additionally, 359 proteins were identified in the DSM fraction (Fig. S1), resulting in a total of 1,578 unique proteins identified (Table S1). The iTRAQ-based quantitative analysis of 740 proteins revealed 65 proteins significantly enriched in the DRM relative to PM (Table 1).

**TABLE 1:** List of DRM-enriched proteins^*a*^

Accession no.	Description (database)	Description (Blast2GO)	E value	Loc.	Functional category	PTM	MW (kD)	TMD	Seq. cov. (uni. pep)
SPRG_19332	Hypothetical protein	C-4 methyl sterol oxidase	1,9E-174	Mem	Lipid Metabolism	Pal, SP	38,5	5	13.9 (4)
SPRG_03506	TKL protein kinase	TKL protein kinase	0	Mem	Signal Transduction	SP	41,5	1	66.6 (19)
SPRG_05981	TKL protein kinase	TKL protein kinase	0	Mem	Signal Transduction	Pal	76,7	2	25 (11)
SPRG_06745	Hypothetical protein	1,3-glucan synthase	0	Mem	Sugar Metabolism	Pal	222,2	16	7.4 (9)
SPRG_21467	Hypothetical protein	1,3-glucan synthase	0	Mem	Sugar Metabolism	Pal	218,9	16	33.2 (50)
SPRG_15364	Hypothetical protein	1,3-glucan synthase	0	Mem	Sugar Metabolism	Pal	246,7	27	32.8 (53)
SPRG_20212	Hypothetical protein	1,3-glucan synthase	0	Mem	Sugar Metabolism	Pal	243,3	27	28.2 (45)
SPRG_09073	Hypothetical protein	1,3-glucan synthase	0	Mem	Sugar Metabolism	Pal	248,0	29	10.6 (16)
SPRG_06591	Hypothetical protein	Cellulose synthase 2	0	Mem	Sugar Metabolism		117,7	8	24.2 (21)
SPRG_10322	Hypothetical protein	Glycoside hydrolase family 72 protein	0	Mem	Sugar Metabolism	SP	61,6	1	20.6 (7)
SPRG_00289	Hypothetical protein	ABC transporter	0	Mem	Transport		108,9	9	17.4 (10)
SPRG_21224	Hypothetical protein	ABC transporter-like protein	0	Mem	Transport		97,2	4	13.6 (8)
SPRG_08695	Hypothetical protein	Amino Acid/Auxin Permease	0	Mem	Transport	Pal	51,7	11	17.6 (6)
SPRG_08708	Hypothetical protein	Amino Acid/Auxin Permease	0	Mem	Transport	SP	49,5	11	14.4 (5)
SPRG_08702	Hypothetical protein	Amino Acid/Auxin Permease	0	Mem	Transport	Pal, SP	50,1	11	9.2 (3)
SPRG_01427	Hypothetical protein	Amino acid-polyamine-organocation family	0	Mem	Transport	Pal	62,7	14	15.5 (7)
SPRG_05017	Hypothetical protein	ATP-binding Cassette superfamily protein	0	Mem	Transport		145,0	12	19.8 (17)
SPRG_07892	Calcium/proton exchanger	Calcium cation antiporter family	0	Mem	Transport	Pal	42,9	11	12 (3)
SPRG_04486	Hypothetical protein	Hypothetical protein	0	Mem	Transport	SP	47,5	5	22.4 (6)
SPRG_06823	Hypothetical protein	Hypothetical protein	0	Mem	Transport	Pal	52,8	11	14.9 (4)
SPRG_02212	Hypothetical protein	Hypothetical protein	0	Mem	Transport		63,3	13	26.9 (15)
SPRG_05749	Hypothetical protein	Hypothetical protein	0	Mem	Transport		62,9	13	15.7 (7)
SPRG_11816	Hypothetical protein	Hypothetical protein	0	Mem	Transport		60,7	13	20.9 (9)
SPRG_15402	Hypothetical protein	Hypothetical protein	0	Mem	Transport		61,8	14	14.9 (7)
SPRG_20997	Hypothetical protein	Major facilitator superfamily protein	0	Mem	Transport		44,6	11	19.6 (7)
SPRG_05443	Hypothetical protein	Major facilitator superfamily protein	0	Mem	Transport		53,8	12	18.7 (6)
SPRG_05444	Hypothetical protein	Major facilitator superfamily protein	0	Mem	Transport		54,9	12	14.7 (4)
SPRG_20105	Hypothetical protein	Major facilitator superfamily protein	0	Mem	Transport		56,7	12	21.3 (8)
SPRG_20110	Hypothetical protein	Major facilitator superfamily protein	0	Mem	Transport		57,3	12	10.2 (5)
SPRG_16977	Hypothetical protein	Major facilitator superfamily protein	0	Mem	Transport	SP	54,0	12	21.3 (7)
SPRG_18136	Hypothetical protein	Major facilitator transporter	2,1E-140	Mem	Transport		22,8	6	24.6 (3)
SPRG_19687	Hypothetical protein	Metal-nicotianamine transporter YSL6-like	0	Mem	Transport		55,9	10	13.7 (6)
SPRG_16564	Hypothetical protein	Metal-nicotianamine transporter ysl6-like	0	Mem	Transport	Pal	73,5	15	23.6 (14)
SPRG_14614	Hypothetical protein	Multidrug ABC transporter ATP-binding protein	0	Mem	Transport		108,9	9	8 (7)
SPRG_08738	Hypothetical protein	Proton-dependent oligopeptide transporter family	0	Mem	Transport	Pal	69,7	14	36.8 (15)
SPRG_20813	Hypothetical protein	Sugar phosphate permease	0	Mem	Transport	Pal	54,6	12	7.2 (4)
SPRG_01434	Hypothetical protein	V-type and A-type ATPase superfamily	0	Mem	Transport		46,6	0	51.3 (18)
SPRG_11570	V-type ATPase	V-type ATPase	8,53E-77	Mem	Transport		13,2	0	49.6 (3)
SPRG_12251	V-type ATPase subunit A	V-type ATPase	0	Mem	Transport		70,2	0	67.3 (33)
SPRG_07890	V-type ATPase subunit B	V-type ATPase	0	Mem	Transport	Pal	56,8	0	79.4 (29)
SPRG_17965	Hypothetical protein	V-type proton ATPase	0	Mem	Transport		56,0	2	9.9 (3)
SPRG_12198	Hypothetical protein	V-type proton ATPase	0	Mem	Transport		97,8	8	40.9 (26)
SPRG_02861	ATP synthase subunit	V-type proton ATPase subunit d 1-like	0	Mem	Transport		45,1	0	78.5 (22)
SPRG_08629	Hypothetical protein	V-type ATPase subunit d 1-like	1,3E-121	Mem	Transport		21,9	0	38.5 (7)
SPRG_08077	Hypothetical protein	V-type ATPase subunit h-like	0	Mem	Transport		51,7	0	49.1 (18)
SPRG_01241	Hypothetical protein	Zinc transporter ZIP1-like	0	Mem	Transport	SP	30,2	8	13 (3)
SPRG_20063	Hypothetical protein	Alpha beta-hydrolase	0	Mem	Unclassified		103,2	8	2.8 (2)
SPRG_03742	Hypothetical protein	Band seven protein	0	Mem	Unclassified	SP	40,2	1	25.4 (7)
SPRG_02955	Hypothetical protein	Band seven protein	0	Mem	Unclassified	SP	36,6	2	37 (9)
SPRG_20522	Hypothetical protein	Cysteine-rich protein	1,5E-86	Mem	Unclassified	GPI, SP	21,2	1	36.3 (5)
SPRG_00390	Hypothetical protein	Hypothetical protein	3,81E-74	Unk	Unclassified	Pal	17,8	0	35.4 (4)
SPRG_20511	Hypothetical protein	Hypothetical protein	1,81E-95	Mem	Unclassified	GPI, SP	22,6	0	32.6 (3)
SPRG_11788	Hypothetical protein	Hypothetical protein	1,72E-84	Mem	Unclassified	GPI, SP	21,9	1	23.3 (5)
SPRG_08418	Hypothetical protein	Hypothetical protein	1,1E-107	Mem	Unclassified	Pal, SP	17,0	2	40.3 (3)
SPRG_07519	Hypothetical protein	Hypothetical protein	2,8E-101	Mem	Unclassified	Pal, SP	16,4	4	14 (2)
SPRG_21249	Hypothetical protein	Hypothetical protein	6,2E-163	Mem	Unclassified		28,7	6	17.1 (3)
SPRG_20171	Hypothetical protein	Hypothetical protein	0	Mem	Unclassified		52,0	6	18.2 (7)
SPRG_05363	Hypothetical protein	Kazal-type proteinase inhibitor	6,82E-64	Mem	Unclassified	GPI, SP	17,7	0	30.5 (3)
SPRG_09559	Hypothetical protein	Kazal-type serine protease	9,8E-115	Mem	Unclassified	GPI, SP	30,6	0	14.4 (3)
SPRG_03147	Hypothetical protein	NC domain-containing protein	0	Unk	Unclassified		46,9	0	30.2 (9)
SPRG_12554	Hypothetical protein	Phosphatidic acid phosphatase	8E-176	Mem	Unclassified	SP	32,2	6	17.6 (5)
SPRG_03215	Hypothetical protein	Spfh band seven phb domain protein	1,7E-132	Mem	Unclassified	SP	37,0	1	15.8 (3)
SPRG_07449	Hypothetical protein	Spfh band seven phb domain protein	0	Mem	Unclassified	SP	37,2	1	26.5 (8)
SPRG_05949	Hypothetical protein	WD-40 repeat-containing protein	0	Unk	Unclassified	Pal	81,8	0	25.5 (11)
SPRG_21598	Hypothetical protein	Yellow stripe like 1 isoform 3	0	Mem	Unclassified	Pal	62,4	11	3.1 (2)

^*a*^ *Loc, cellular location; Mem, membrane-bound; Unk, unknown; Seq. Cov.,percent sequence coverage; uni. pep., number of unique peptides identified; PTM, predicted post-translational modifications; Pal, palmitoylation; SP, signal peptide; GPI,glycosylphosphatidylinositol; TMD, number of transmembrane domains.*

### Predictions of cellular localization and biological process

**Fig 3:**
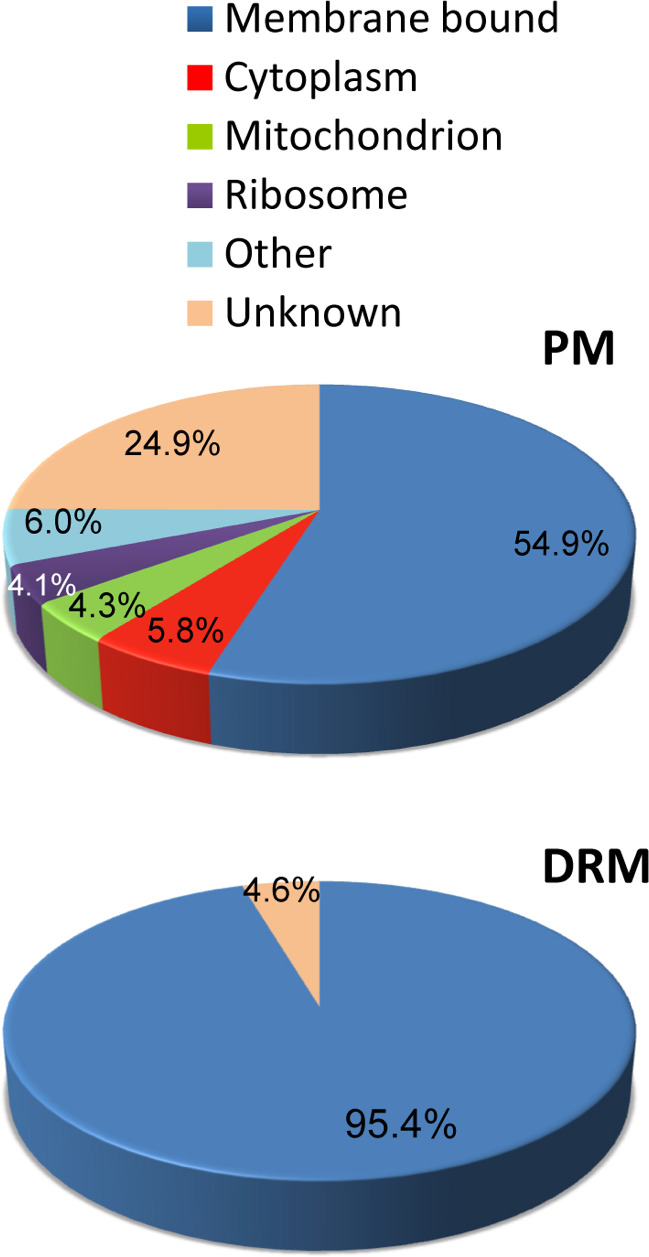
Predicted cellular localization of proteins identified after in-solution digestion and MS/MS analysis of PM and DRM fractions. PM, plasma membrane; DRM, detergent-resistant microdomains. Gene Ontology information was obtained using Blast2GO.

Approximately, 55% of the proteins identified in the PM was classified as membrane bound. However, with the exception of 4.6% of proteins with unknown localization, all DRM-enriched proteins were predicted to be membrane bound (Fig. 3). The remaining proteins in the PM were classified as cytoplasmic (5.8%), mitochondrial (4.3%), and ribosomal (4.1%), together with many proteins of unknown localization (24.9%) (Fig. 3). Our results reflect both the challenges of achieving a complete PM purification, which often co-purifies with endoplasmic reticulum and other cytoplasmatic components, and the enrichment in membrane-bound proteins following Triton extraction of PM preparations.

**Fig 4:**
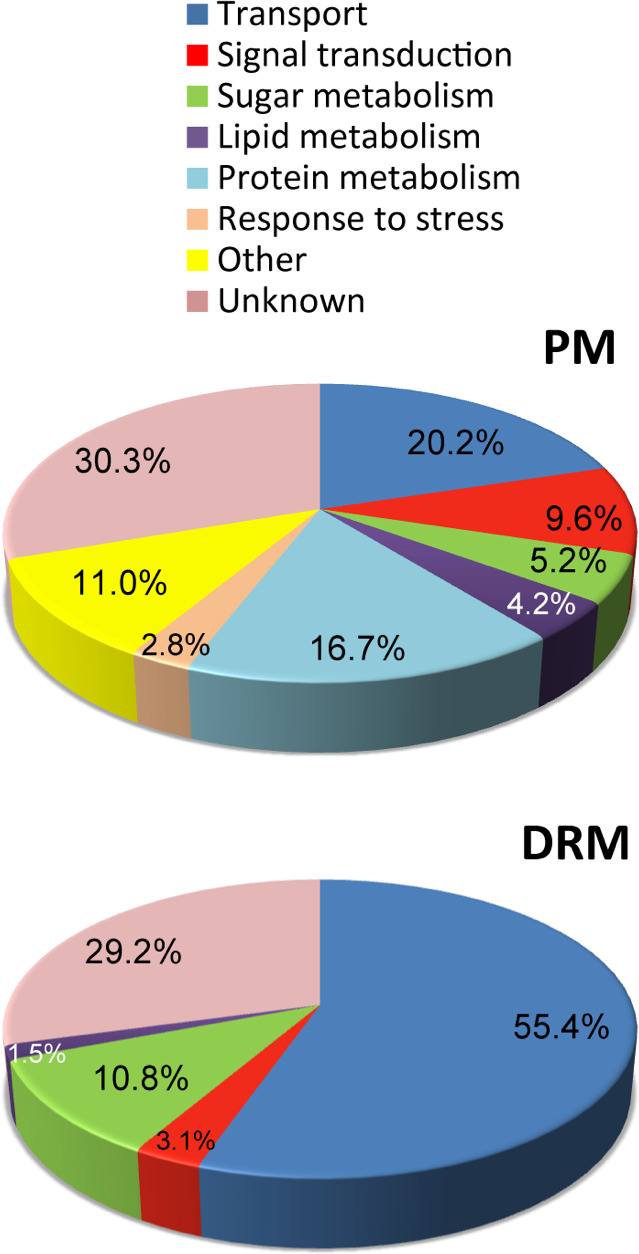
Predicted functional classification of proteins identified after in-solution digestion and MS/MS analysis of PM and DRM fractions. PM, plasma membrane; DRM, detergent-resistant microdomains. Gene Ontology information was obtained using Blast2GO.

As expected, the proteins identified in the PM were found to be associated with a wide range of biological processes (Fig. 4). Established PM functions including transport and signal transduction were well represented in our study, corresponding to 20.2% and 9.6% of the total proteins, respectively. Additionally, 16.7% of the proteins identified was associated with protein metabolism while nearly a third (30.3%) was unknown or unclassified. Sugar and lipid metabolisms represented nearly 10% of the total proteins (5.2% and 4.2%, respectively). Interestingly, proteins identified in the DRMs are associated with far fewer functions (Fig. 4). The highest represented categories were transport (55.4%) and sugar metabolism (10.8%). The transporters can be further categorized into groups including the V-type ATPases (25%), major facilitators (19.4%), ABCs (11.1%), and amino acid transporters (11.1%) (Table 1). Some metal transporters and a calcium antiporter were also identified as enriched in the DRMs (Table 1). Interestingly, all DRM-enriched proteins grouped under sugar metabolism are associated with glucan biosynthesis and/or remodeling (Table 1). Five of the seven proteins in this category were identified as β-1,3-glucan synthases with the remaining two being a β-1,4-glucan synthase and a β-1,3-glucanosyltransglycosylase (GH72 family) (Table 1). It is noteworthy that among the proteins of unknown-unclassified function (~29%), many corresponded to Band 7-type proteins (Table 1), which are established DRM markers in other types of eukaryotic cells (48). The functions of these proteins are just beginning to be elucidated, but roles in the formation of membrane microdomains and associated processes, including endocytosis and mechanosensation, have been proposed (48).

### Biochemical features of DRM-enriched proteins

**Fig 5:**
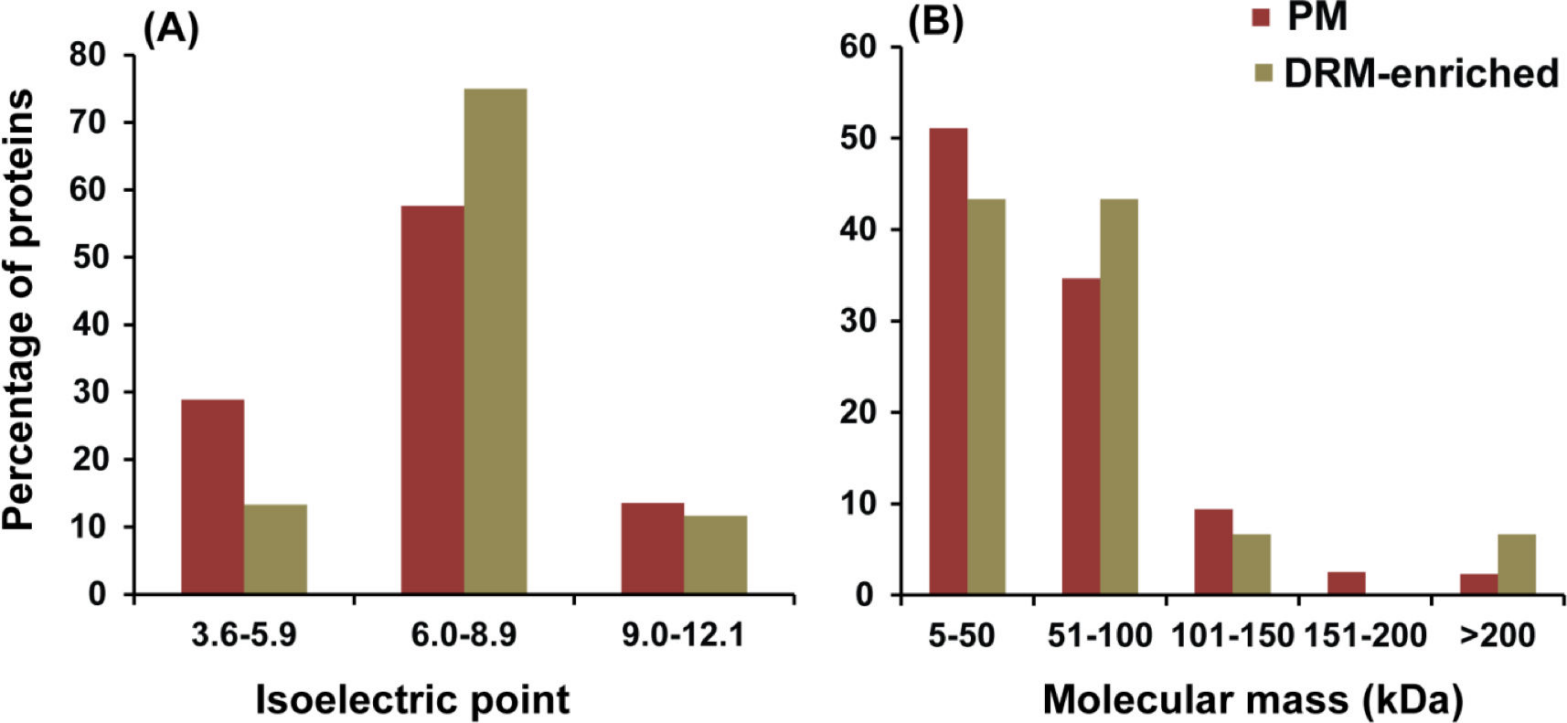
Biochemical features of DRM-enriched (green bars) and total plasma membrane proteins (red bars). (**A**) Isoelectric point; (**B**) molecular mass.

Relative to the PM, a higher percentage of the proteins in the DRM exhibited a neutral isoelectric point (pI 6.0–8.9), 58% and 75%, respectively (Fig. 5A). In contrast, acidic proteins (pI < 6) were 16% less present in the DRM as compared with the PM. Similar percentages of alkaline proteins (pI > 8.9) were identified in the microdomains and the total PM. On average, higher molecular masses were observed for DRM-enriched proteins (Fig. 5B), a trend largely attributed to the 9% increase in proteins ranging between 51 and 100 kDa (34% PM vs. 43% DRM). Additionally, 6.7% of the DRM proteins exhibited molecular weights over 200 kDa, compared with the 2.3% observed in the PM.

**Fig 6:**
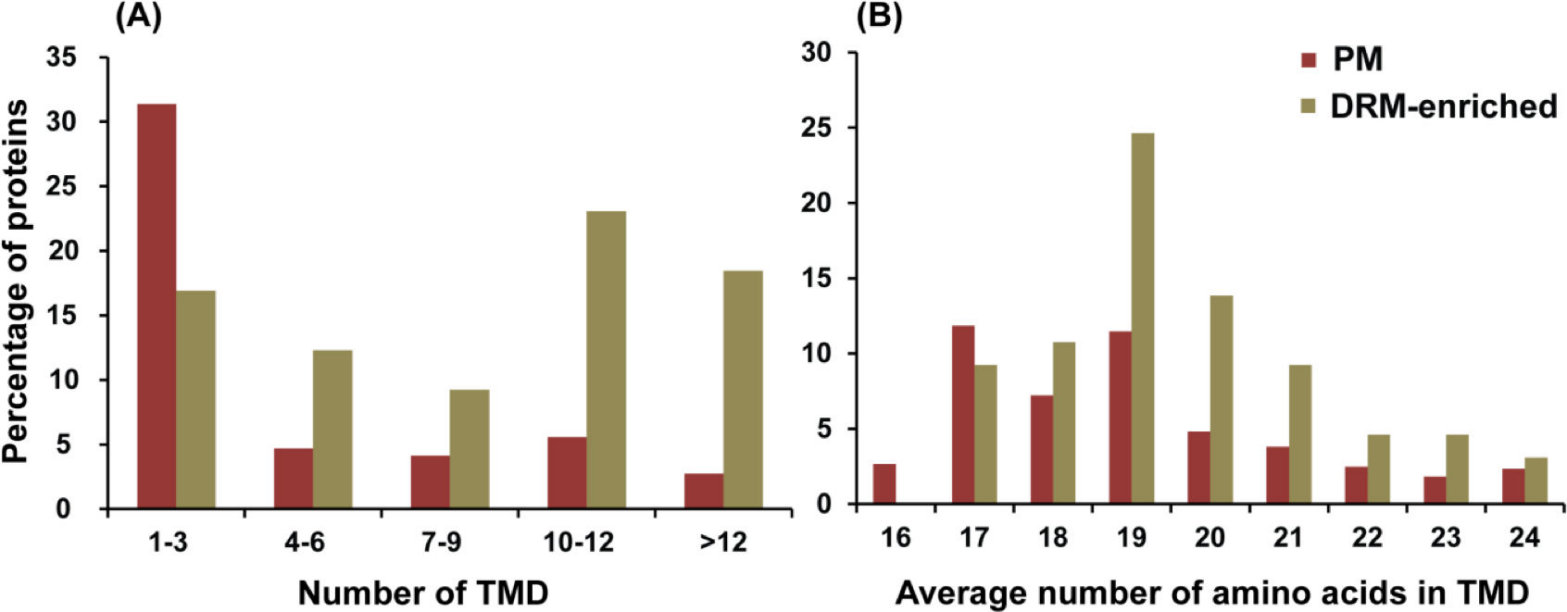
Analysis of the properties of TMD in PM proteins. DRM-enriched (green bars) and total PM proteins (red bars). (**A**) Number of TMD; (**B**) average number of amino acids in TMD.

Only 7 of 65 (10.8%) DRM-enriched proteins did not contain any TMD and/or membrane-anchoring acylation sites (Table 1). Furthermore, relative to the PM proteins, the DRM-enriched proteins were far more likely to contain four or more transmembrane domains, 17.1% and 63.1%, respectively (Fig. 6A). Additionally, the DRM-enriched proteins were found to contain a higher average number of amino acids per TMD (Fig. 6B). While 26.7% of proteins in the PM contained more than 18 amino acids per TMD, this percentage raised to 59.9 in the case of DRM-enriched proteins (Fig. 6B), supporting the previous observation that DRMs are thicker compared with the detergent-soluble part of the PM (23).

### β-1,3-glucan synthases are located in the DRMs

**Fig 7:**
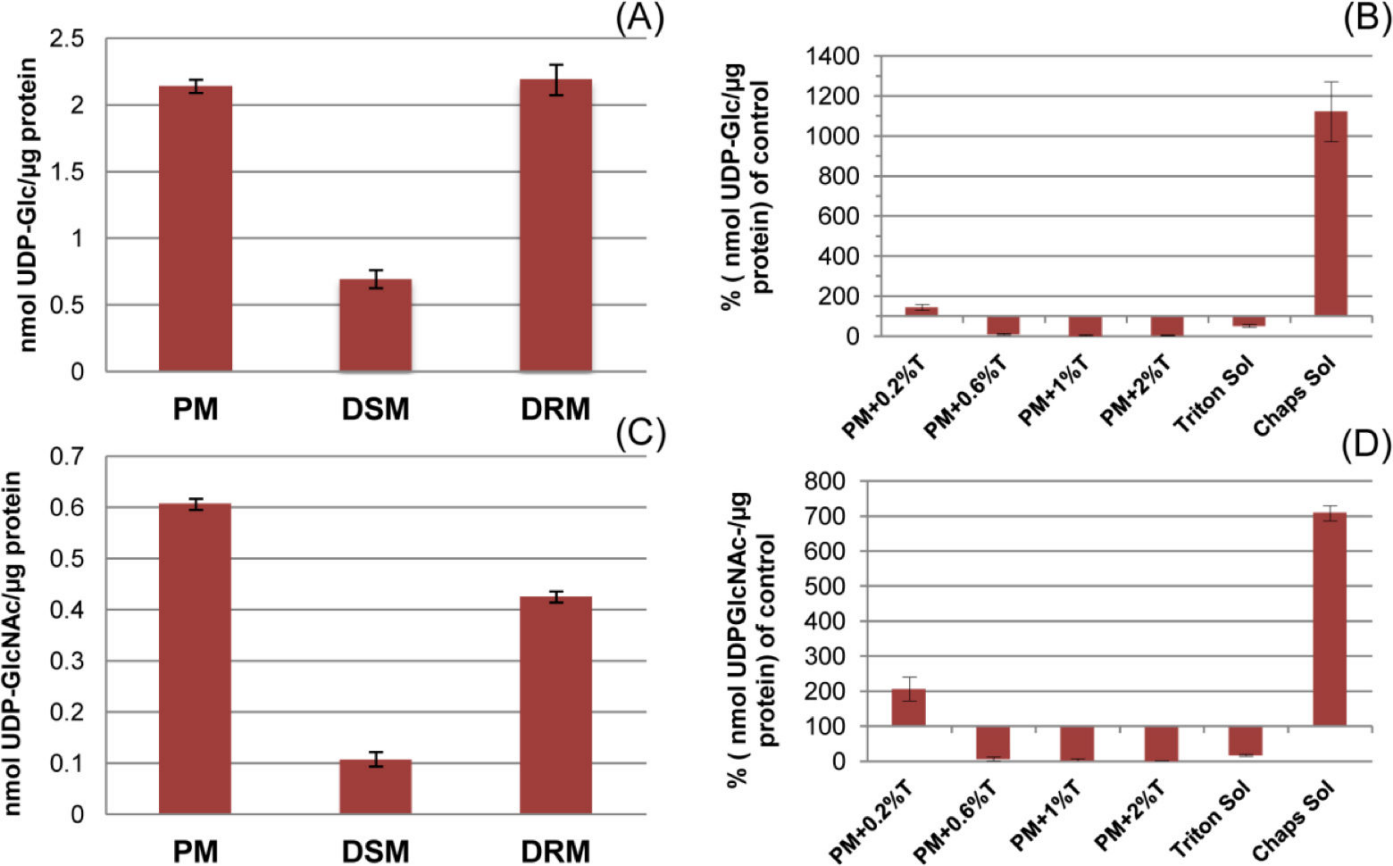
Carbohydrate synthase activities of proteins from PM, DRM, and DSM fractions. PM, plasma membrane; DRM, detergent-resistant microdomains; DSM, detergent-soluble membranes. (**A and B**) Glucan synthase assays; (**C and D**) chitin synthase assays. “T” in panels B and D states for Triton X-100.

Previous work in the species *S. monoica*, a close relative of *S. parasitica*, demonstrated via enzymatic assays an enriched presence of cell wall polysaccharide synthase activities (chitin and β-1,3-glucan synthases) in the DRMs (19). Together with the high enrichment of carbohydrate synthases in the *S. parasitica* DRMs observed in our study, these findings encouraged us to investigate glycosyltransferase activities in the DRM further. Chitin and β-1,3-glucan enzymatic activities were measured using radioactive nucleotide-sugars as substrates and quantifying the incorporation of radiolabeled monosaccharides into insoluble carbohydrates by the proteins in the PM, DRM, and DSM fractions (Fig. 7). Relative to the PM, glucan and chitin synthase activities in the Triton extracts (DSM) were reduced by 67% and 83%, respectively (Fig. 7A and C). The decrease in chitin synthase activity was comparatively modest in the Triton-resistant membranes (Fig. 7C), and the glucan synthase activity was slightly higher in this fraction (Fig. 7A). The negative effect of Triton X-100 on the activity of some glycosyltransferases has been shown (41). Therefore, to determine the effect of Triton X-100 on our target proteins, we performed assays with increasing concentrations of Triton X-100 added to the reaction mixtures (Fig. 7B and D). Our results indicate that Triton X-100 concentrations exceeding 0.2% almost completely inhibit glucan and chitin synthase activities in the *S. parasitica* PM. It was further shown that the Triton-solubilized PM had much lower activity levels than the non-solubilized PM control. In contrast, CHAPS solubilization improved enzymatic activities (11- and 7-fold for glucan and chitin synthase, respectively). Although challenging to verify, our data suggest that the Triton X-100 incubations required for DRM preparation negatively affect DRM-glycosyltransferase activities. It is therefore probable that DRMs in their native state may be enriched in glycosyltransferase activity, as is supported by our proteomic results.

### Sequence analysis and expression levels of the *S. parasitica* β-1,3-glucan synthases

**Fig 8:**
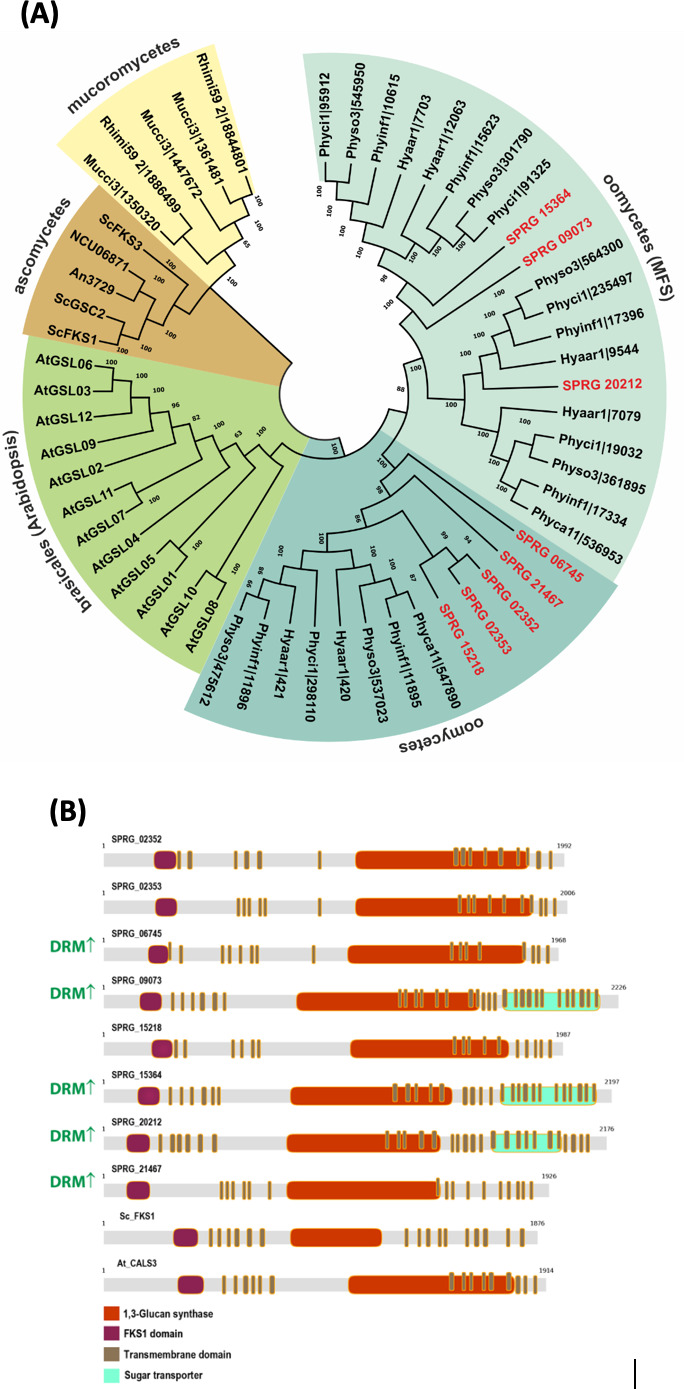
Sequence analysis of the *S. parasitica* β-1,3-glucan synthase genes. (**A**) Phylogenetic tree showing different groups of glucan synthases from oomycetes: *S. parasitica* CBS 223.65 (SPRG, highlighted in red), *P. infestans T30-4* (*Phyinf*), *P. cinnamomi* var *cinnamomi* (*Phyci1*), *P. sojae* (*Physo3*), *Phytophthora capsica LT1534* (*Phyca11*), and *H. arabidopsidis Emoy2* (*Hyaar1*)); ascomycetes: *S. cerevisiae* [(45), *ScFKS1, ScFKS2, ScGSC2*], *A. nidulans* (*AN3729*), and *N. crassa* (*NCU06871*); mucoromycetes: *M. lusitanicus* (*Mucci3*) and *R. microsporus* (*Rhimi59 2*); and Brassicales: *A. thaliana* [AtGSL, (46)]. (**B**) Domain comparison. β-1,3-Glucan synthase genes from *S. cerevisiae* (*Sc_FKS1*) and *A. thaliana* (*At_CALS3*) are also represented for comparison. Proteins enriched in the DRM are labeled at the left end as DRM↑.

The amino acid sequences from the eight full-length β-1,3-glucan synthases predicted in the *S. parasitica* genome were aligned and compared with sequences available from five plant pathogenic oomycetes species (*Phytophthora infestans*, *Phytophthora cinnamomi*, *Phytophthora sojae*, *Phytophthora capsici*, and *Hyaloperonospora arabidopsidis*) and three and two representatives from the ascomycetes [*Saccharomyces cerevisiae* (45), *Aspergillus nidulans*, and *Neurospora crassa*] and mucoromycetes (*Mucor lusitanicus*, *Rhizopus microsporus*), respectively, and with one representative from plants (Brassicales), *Arabidopsis thaliana* (46). Phylogenetic analysis of *S. parasitica* and the other oomycete β-1,3-glucan synthases indicated a higher similarity with the plant orthologs than with fungi (Fig. 8A). Within the oomycetes, the β-1,3-glucan synthases were separated into two distinct groups. In contrast to the first group, which harbors only the β-1,3-glucan synthase domain, proteins in the second group contain an additional major facilitator sugar transporter domain (MFS) at the C-terminus (Fig. 8B; SPRG_15364T0, SPRG_09073T0, SPRG_20212T0). This MFS domain seems to be conserved in oomycetes, but to our knowledge, sugar transporter domains within β-1,3-glucan synthases have not been described in any other phylum. It is interesting that all three proteins were highly enriched in the DRMs.

Additionally, our proteomic results aligned with RNAseq gene expression data from the *Saprolegnia genome* Sequencing Project (Broad Institute of Harvard and MIT; http://www.broadinstitute.org/) and from Jiang et al. (49). These data confirmed measurable gene expression levels for all 65 proteins enriched in the DRM and identified in the PM [Table S6 and (49)]. Furthermore, we showed that five of the six most highly expressed β-1,3-glucan synthases are enriched in the DRMs (Fig. S2).

## DISCUSSION

Although oomycetes grow in a mycelial form similar to true fungi, they are classified in a different taxonomic group, the Stramenopiles, with diatoms and brown algae (50). Furthermore, despite sharing many biochemical features, oomycetes and fungi can be biochemically distinguished by their cell wall polysaccharide compositions (4, 50, 51). The similarities between oomycetes and plants, including their phylogenetic relationship and cellulose-dominated cell walls (4, 52), are intriguing, and we can further our understanding of their relationship by comparing their DRM composition.

*In silico* functional analyses of the *S. parasitica* DRM-enriched proteins led to the identification of two main functional categories, transport (55.4%) and sugar metabolism (10.8%). The relevance of proteins present in each category and their association with the DRMs is discussed below.

### V-Type proton ATPases

Several types of transport-related proteins were identified as enriched in the DRMs of *S. parasitica*. V-type proton ATPases were the most prominent group (9 of 65 proteins). Likewise, the A and B subunits of V-ATPases were among the few proteins previously identified in the *S. monoica* DRMs (19). Six and five of these V-ATPases were further expressed in the mycelium of *S parasitica* but downregulated in the cyst or germinating cyst, respectively (49). These findings were also confirmed by quantitative proteomic enrichment in the mycelial growth stage of *S. parasitica* (53). Interestingly, V-type ATPases are also recognised constituents of animal (54) and plant (55–57) DRMs. In the model system *S. cerevisiae*, the plasma membrane proton ATPase Pma1p (comparable to V-ATPases) is considered a DRM marker (58). Similarly, proteomic characterization of the human pathogenic fungus *Candida albicans* identified the ATPase Pma1p as 1 of the 29 proteins found in the DRM (59). Pma1p has been well characterized in yeast cells. It is thought to play a vital role in maintaining ion homeostasis by creating an electrochemical gradient of protons across the plasma membrane. Therefore, it is possible that Pmalp similarly maintains ion homeostasis in *S. parasitica*.

### Glucan synthesis

β-1,3-Glucans represent 25% of the polysaccharide cell wall content and play a major structural role in the mycelial cell wall of *S. parasitica* (4). Due to their relatively large size (up to 250 kDa) and the high number of transmembrane helices they carry (16–29 TMDs), the β-1,3-glucan synthases are challenging to characterize using biochemical approaches. Of the eight predicted β-1,3-glucan synthases in the *S. parasitica* genome, the seven with detectable levels of gene expression in the mycelium were identified in our study to reside in the PM. Furthermore, five of these seven proteins were enriched in the microdomains, suggesting that the microdomains may provide specialized “hubs” for synthesizing this cell wall component. Biochemical activity assays have confirmed this finding, and these results align with previous work completed in *S. monoica* (19). Likewise, these glucan biosynthetic proteins are a component of plant DRMs (24, 29, 56, 57). However, in plants, glucans appear to serve different functions, are present in small quantities, and are only found in specialized locations at particular stages of growth and differentiation (60). For example, it has been suggested that glucans in plant DRMs form plugs in response to stress (24). In addition, glucans are thought to play a structural role in the cell walls of oomycetes. Despite β-1,3-glucans being abundant cell wall components of most, but not all fungal species (51, 61), their biosynthetic enzymes have not been shown to be enriched in DRM. However, some similarities exist between oomycete and fungal DRMs not shared with plants. Specifically, the β-1,3-glucanosyltransferase found in *S. cerevisiae* DRMs [GAS1; (23)] is similar to the GH72 found in *S. parasitica*, but no similar protein has been identified in plant DRMs.

In *S. parasitica*, β-1,4-glucans are more abundant than β-1,3-glucans, representing approximately 50% of the cell wall polysaccharides (4). However, biochemical assays did not detect any cellulose synthase activity in the *Saprolegnia* DRM (19). Contradicting these data, our proteomics approach did show that one of the four cellulose synthases found in the PM is enriched in DRM (SPRG_06591T0) (Table 1). Interestingly, quantitative real-time polymerase chain reaction data indicate that the cellulose synthase identified in the DRM has the lowest expression level in the mycelium (62). It is possible that location in the microdomains, rather than distribution across the PM, contributes to comparatively low expression levels of this specific enzyme. In plants, cellulose synthases form complexes that synthesize β-1,4-glucan microfibrils (63). But whether oomycete cellulose synthases are organized in similar large complexes remains elusive, although one report showed the co-immunoprecipitation of several cellulose synthase subunits in *Phytophthora capsica* (64). However, our data suggest that the cellulose synthase identified in the DRM is not physically associated with the other isoforms.

### Major facilitator transporters

Seven MFSs were identified, representing 10% of all DRM-enriched proteins found. Despite being one of the two largest families of membrane transporters identified across all species, MFSs (65, 66) have only been reported as being present in the DRMs in two cases. In poplar cell suspension cultures, major facilitator transporters represented only 1% of the total proteins identified (24). In *C. albicans*, two out of three quinidine drug resistance MFS transporters were detected in DRMs and contained putative palmitoylation sites commonly observed in proteins localized to DRMs (67).

An outstanding finding of this study is the presence of three DRM-enriched β-1,3-glucan synthases that contain an MFS as an additional domain, with predicted sugar transport activity (Fig. 8). In all three cases, the MFS domains are located after the 1,3-glucan synthase biosynthetic domain, close to the C-terminus, and are membrane anchored by means of several transmembrane helices (7 to 12 TMD). Interestingly, genome profiling identified these domains as unique to oomycete β-1,3-glucan synthases including multiple species of the oomycete genera *Saprolegnia*, *Aphanomyces*, *Phytophthora*, and *Albugo*, but these domains have not been found in the glucan synthases of other eukaryotes. Further experimentation is required to improve our understanding of the role of these domains in glucan biosynthesis. It is tempting to speculate that they contribute to the extrusion of the newly synthesized glucan chains across the PM (68).

### ABC transporters

A second well-represented transporter superfamily found in the DRMs of *S. cerevisiae*, poplar, and *S. parasitica* is the ABCs (24, 69). The ABCs are transmembrane proteins that utilize ATP-binding energy and hydrolysis to transport various substrates across the PM including metabolic products, lipids, sterols, and drugs (70). This transporter type is recognised to be highly represented in plant pathogens, including plant pathogenic oomycetes (71). In entomopathogenic pathogens, duplication events in subclasses of ABC Transporters increased their numbers (72).

MFSs and ABCs are thought to facilitate the removal of host defense toxins in fungal and oomycete pathogens through efflux pumps (73). The predominance of MFSs and ABCs in the *S. parasitica* DRM supports the possibility of a role for these microdomains in pathogenesis.

### Amino acid transporters

Four amino acid transporters were identified in the *S. parasitica* DRM, three of which were members of the eukaryotic-specific amino acid/auxin permease family. Amino acid transporters are not well represented in plant DRMs [only one in poplar (24)], but several reports indicate their presence in *Saccharomyces* DRMs (27, 74, 75). Interestingly, although the genome of *S. parasitica* contains 56 predicted amino acid transporters, less than 20 have orthologs or closely related paralogs in other oomycete genomes (49), perhaps in part explaining their relative abundance in the DRMs.

In addition to the mycelium, there are several other developmental stages in the *S. parasitica* life cycle (3). Sporangia form at the end of hyphal cells and release many motile zoospores, which swim, encyst, and either germinate or, if no tissue is suitable for germination, release secondary zoospores (3). The pre-infection stage is the stage before host tissue colonization, in this case the germinating cyst (49). Interestingly, RNAseq data on mycelium, cysts, and germinating cysts (49) indicate that the expression of the DRM-enriched proteins is largely downregulated in the cysts relative to the mycelium (25 proteins in cysts and 17 in germinating cysts; Table S6). Only two DRM-enriched proteins (SPRG_05017-ABC transporter and SPRG_20171-unknown function) were overexpressed in the encysted stages. These data suggest that DRMs are not involved in encystment or infection mechanisms. However, as mentioned previously, DRMs are putatively involved in cell wall biosynthesis and carry efflux pumps, perhaps combating intracellular toxin accumulation. Therefore, we propose that DRMs may play a role in the later stages of pathogenesis when the mycelium spreads over the infected tissues.

In conclusion, this study provides the first plasma membrane proteome analysis in oomycetes. We have identified 65 proteins enriched in microdomains. *In silico* analysis of the DRM-enriched proteins allowed us to further describe them as having higher molecular masses and greater numbers of TMDs, relative to PM proteins. Interestingly, the *S. parasitica* DRM includes proteins involved in molecular transport and β-1, 3-glucan synthesis, putatively supporting pathogenesis. The knowledge gained from this work holds promise for aiding the development of disease control measures targeting specific proteins of *S. parasitica*. Although inhibitors targeting various fungal cell wall polysaccharide synthases have been identified, their exploration in oomycetes remains limited. Our data provide a basis for selecting potential inhibitor targets within the PM, which will facilitate the development of a screening pipeline aimed at identifying novel targets and inhibitors.

## SUPPLEMENTAL MATERIAL

Supplemental material spectrum.00348-24-s0001.pdf Fig. S1 and S2.

Table S1 spectrum.00348-24-s0002.xlsx Total protein list from all replicates.

Table S2 spectrum.00348-24-s0003.xlsx Protein list from first biological replicate.

Table S3 spectrum.00348-24-s0004.xlsx Protein list from second biological replicate.

Table S4 spectrum.00348-24-s0005.xlsx Protein list from third biological replicate.

Table S5 spectrum.00348-24-s0006.xlsx Protein list identified from the soluble fraction.

Table S6 spectrum.00348-24-s0007.xlsx Comparison of DRM-enriched proteins with expression levels of their corresponding genes.

ASM does not own the copyrights to Supplemental Material that may be linked to, or accessed through, an article. The authors have granted ASM a non-exclusive, world-wide license to publish the Supplemental Material files. Please contact the corresponding author directly for reuse.
