# Supplementary material for: Quantitative proteomic analysis of plasma membranes from the fish pathogen Saprolegnia parasitica reveals promising targets for disease control
Source: Microbiol Spectr. 2024 Jun 18;12(8):e00348-24. doi: 10.1128/spectrum.00348-24 (PMC11302233; doi:10.1128/spectrum.00348-24)
Supplement: Supplemental material — Fig. S1 and S2. [file spectrum.00348-24-s0001.pdf]

Quantitative proteomic analysis of plasma membranes from the fish pathogen *Saprolegnia parasitica* reveals promising targets for disease control

Hugo Mélida, Lisa Kappel, Sadia Fida Ullah, Vincent Bulone, Vaibhav Srivastava

Supplemental Material: This article contains Supplemental Figs. S1 and S2, and Tables S1-S6.

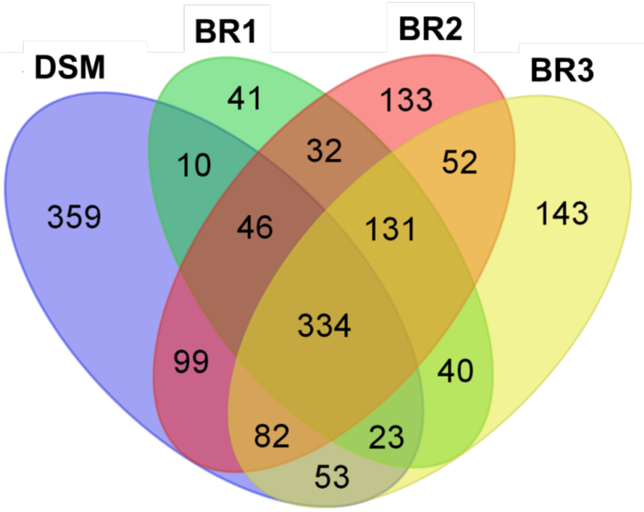

Figure S1. Qualitative proteomic analysis of plasma membrane (PM), detergent-resistant microdomains (DRM) and detergent-soluble membranes (DSM). The Venn diagram represents the number of proteins identified from in-solution digestion and MS/MS analysis of PM and DRM fractions for each of the three biological replicates (BR). In total, 657, 909 and 858 proteins were identified by MS/MS analysis of BR1, BR2 and BR3, respectively. Additionally, 1006 proteins were identified by MS/MS analysis of the DSM fraction.

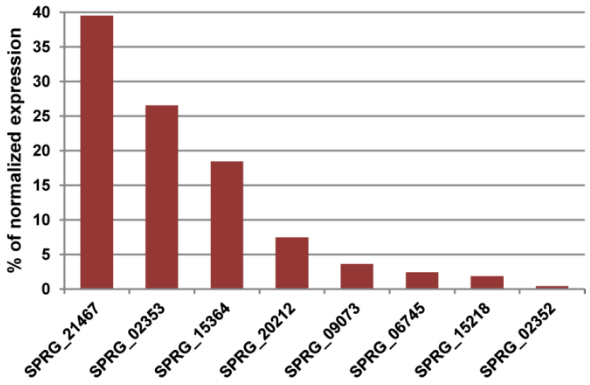

Figure S2. Expression profiles of β-1,3-glucan synthase genes. Transcript abundance normalized to 100% from RPKM (reads per kb per million reads) normalized values. RNAseq information was retrieved from "The *Saprolegnia genome* Sequencing Project, Broad Institute of Harvard and MIT (http://www.broadinstitute.org/)".

Supplemental Tables:

Table S1: List of all proteins identified by MS/MS from three biological replicates of PM, DRM and DSM samples

Table S2: List of all unique proteins (together with their corresponding peptides) identified by iTRAQ experiments from the first biological replicate of DRM and PM samples

Table S3: List of all unique proteins (together with their corresponding peptides) identified by iTRAQ experiments from the second biological replicate of DRM and PM samples

Table S4 List of all unique proteins (together with their corresponding peptides) quantified by iTRAQ experiments from the third biological replicate of DRM and PM samples

Table S5: List of all unique proteins together with corresponding peptides, identified from Detergent-Soluble Membrane (DSM) samples

Table S6: Comparison of DRM-enriched proteins (Table 1) with the expression levels of their corresponding genes in *Saprolegnia* cyst and germinating cyst stages, relative to mycelium [49]
